# A dominant SRCAP truncating mutation promotes squamous cell carcinoma progression

**DOI:** 10.1038/s41389-025-00576-z

**Published:** 2025-08-26

**Authors:** Stephenie H. Droll, Elena I. O. Dewar, Celia Xue, Max C. Levine, Benny J. Zhang, Xiaomin Bao

**Affiliations:** 1 Northwestern University, Department of Molecular Biosciences, Evanston, IL 60208 USA; 2 Northwestern University, Department of Dermatology, Chicago, IL 60611 USA; 3 Robert H. Lurie Comprehensive Cancer Center, Northwestern University, Chicago, IL 60611 USA

**Keywords:** Cancer genetics, Mutation

## Abstract

The majority of life-threatening cancers arise from epithelial tissues. These epithelial cancers include cutaneous squamous cell carcinoma (cSCC), the second-most common cancer. cSCC is highly invasive and accounts for an estimated 15,000 deaths each year. We identified SRCAP, a chromatin remodeler that regulates the chromatin occupancy of the histone H2A variant H2A.Z, as a frequently mutated gene in cSCC. Analysis of cSCC mutations in epithelial cancers identified a hotspot truncating mutation in SRCAP, which removes 42% of the protein sequences after amino acid 1879. While SRCAP mutations have been previously connected to the pathogenesis of Floating-Harbor syndrome (FHS), these typically occur downstream, with a hotspot mutation leading to protein truncation after amino acid 2444. We found that expressing the SRCAP-1879 truncation in an HRas-CDK4-driven cSCC model was sufficient to increase proliferation, impair terminal differentiation, and accelerate invasion. Mechanistically, the expression of SRCAP-1879 in primary human keratinocytes was sufficient to dysregulate genes crucial for carcinogenesis (e.g., proliferation, differentiation, and motility) without altering H2A.Z occupancy. In particular, the expression of SRCAP-1879 truncation led to strong induction of the matrix metalloproteinase MMP9 expression level, accompanied by increased keratinocyte cell motility, which was sensitive to matrix metalloprotease inhibition. In contrast, the expression of the SRCAP-FHS truncation did not increase but instead reduced cell motility as well as the expression of MMP9. Taken together, our findings identify a previously under-characterized role of the SRCAP-1879 truncating mutation in promoting multiple aspects of epithelial cancer progression, including invasion, distinct from the well-recognized roles of SRCAP mutations in FHS pathogenesis.

## Introduction

Tumors occurring in epithelial tissues account for greater than 85% of all cancers [1]. Cutaneous squamous cell carcinoma (cSCC) arises from the skin epidermis and is the second-most common cancer worldwide. Although cSCC is not included in national cancer registries, about 700,000 cases are diagnosed annually in the United States, and the incidence of cSCC continues to rise [2]. Surgical removal of the tumor is frequently curative, but roughly 5% of cases will metastasize, killing about 15,000 people per year, which means cSCC causes more annual deaths than melanoma [3].

Skin is the body’s primary physical and immunological barrier against the environment. The outermost portion of skin, the epidermis, is composed of keratinocytes. In healthy epidermis, proliferative cells reside in the basal layer and are separated from the dermis by the basement membrane. Differentiating keratinocytes migrate toward the skin’s surface, cease proliferation, and activate genes to produce the epidermal barrier [4].

Ultraviolet radiation drives mutation in morphologically normal skin at a rate greater than many solid tumors. Therefore, the resulting keratinocyte carcinomas, cSCC and basal cell carcinoma (BCC), possess the highest mutational burdens among all cancers [5–7]. Initially, keratinocyte clones containing mutations in drivers such as TP53, NOTCH1, or RAS expand unnoticed [8–10]. Additional mutations drive progression from clonal proliferation to premalignant lesions to cSCC, and this extreme mutational burden impedes efforts to identify cSCC drivers and to develop therapeutics.

SRCAP is a chromatin remodeler that incorporates the histone H2A variant, H2A.Z, into nucleosomes. SRCAP forms a large, multiprotein complex that is essential for embryonic development [11–13] and also participates in processes including DNA repair, chromosome segregation, lineage commitment, and differentiation [14–17]. The rare developmental disease, Floating Harbor Syndrome (FHS), is caused by dominant, heterozygous, germline truncating mutations in SRCAP, with a hotspot mutation truncating about 24% from the C terminus after amino acid 2444 in exon 34 [18]. FHS Patients are characterized by developmental delays, distinctive facial features, and intellectual disability, with no strong evidence suggesting increased cancer risk. However, emerging reports indicate that SRCAP may play a role in cancer initiation and progression [19–21], but additional research is needed as even its status as a tumor suppressor or oncogene remains unclear. Considering the large size of the SRCAP protein, with a total of 3230 amino acids, it remains to be established whether other mutations than the ones involved in FHS can influence cancer progression.

Using data from cBioPortal [22], we identified SRCAP as a frequently mutated gene in cSCC and identified a mutational hotspot in SRCAP using pan-cancer data. This hotspot mutation truncates the SRCAP protein after amino acid 1879. Expression of this truncated form of SRCAP in primary human keratinocyte dysregulates the expression of proliferation and differentiation genes and strongly induces the expression of MMP9, accompanied by increased cell motility. Expressing this truncated form of SRCAP in an HRas-CDK4-driven cSCC model increased proliferation, impaired differentiation, and increased dermal invasion. Matrix metalloprotease (MMP) inhibitors reduce SRCAP-mutation-induced migration. However, the expression of the SRCAP-FHS truncation did not increase, but rather reduced cell migration and MMP9 expression. Our findings suggest that the SRCAP-1879 hotspot truncation represents a novel driver of epithelial cancers such as cSCC, distinct from the impact of the truncating mutation previously identified in FHS.

## Results

### SRCAP is recurrently mutated in keratinocyte carcinomas and other cancer types

**Fig. 1:**
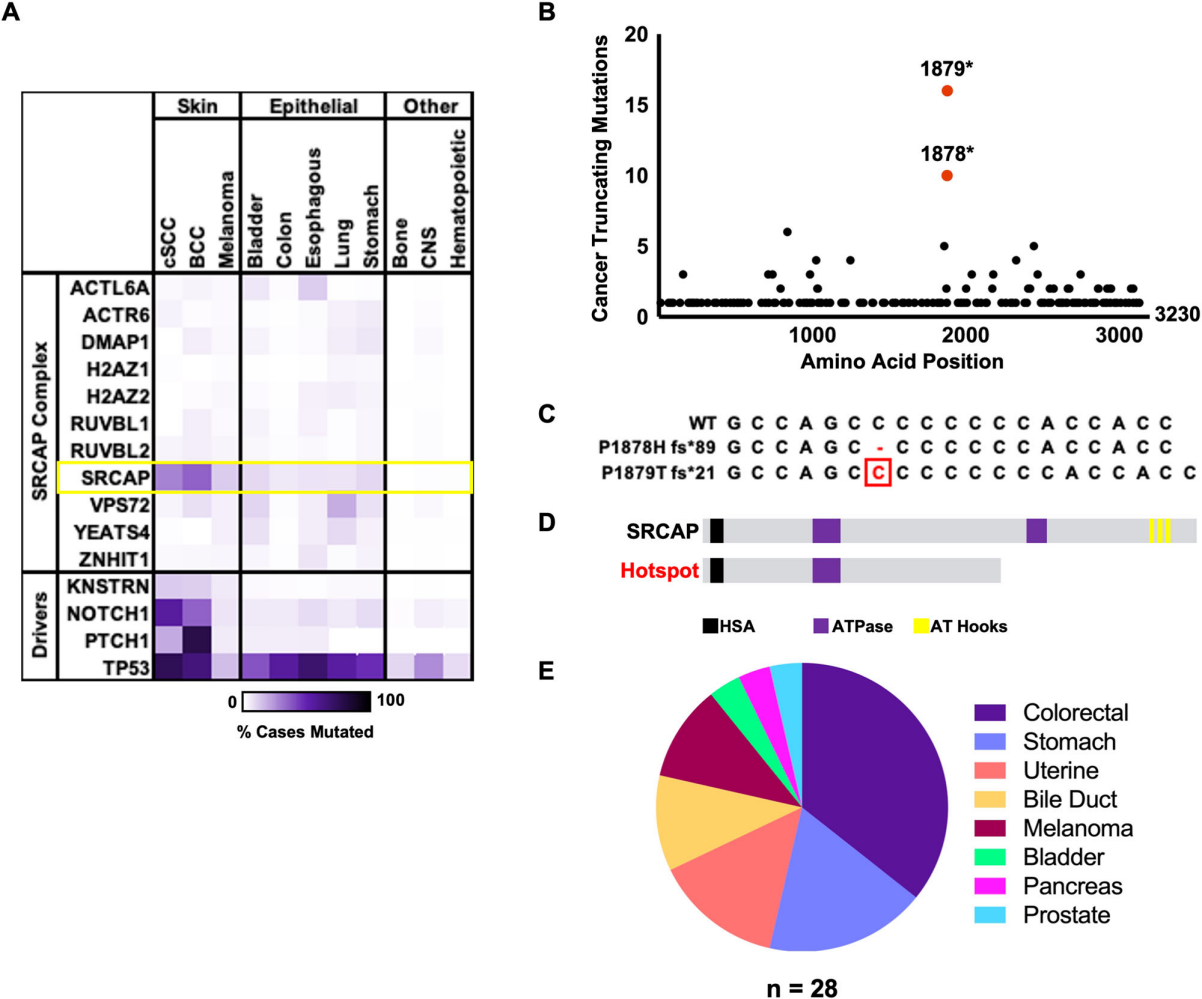
SRCAP is recurrently mutated in cancer. **A** Heatmap showing the mutation frequency of the components of the SRCAP complex and known cancer drivers. **B** Plot showing the frequency of truncating mutations at each amino acid of SRCAP in pan-cancer data. Red dots indicate the mutational hotspot. **C** Alignment of the DNA sequences at the mutational hotspot. **D** Diagram of wild-type and hotspot truncation protein structure. **E** Pie chart showing tissue of origin associated with the hotspot mutations.

We began our investigation by retrieving mutation data from the cBioPortal database for each component of the SRCAP chromatin remodeling complex. SRCAP is the only component of the complex that is frequently mutated. Further, SRCAP is most frequently mutated in keratinocyte carcinomas (Fig. 1A). SRCAP is in the top 2% most frequently mutated genes in cSCC, which is the more aggressive of the two keratinocyte carcinomas. Because relatively few cases of cSCC (176 patients) have been sequenced for mutations, we used pan-cancer data (>91,000 patients), which revealed that missense and truncating mutations occur across the length of SRCAP. Given the sheer volume of mutations, we focused on truncating mutations because of their obvious deleterious effects. When considering pan-cancer truncating mutations, a mutational hotspot affecting amino acid position 1878 or 1879 emerges (P1878H fs*89 and P1879T fs* 21). (Fig. 1B). The DNA sequence at this hotspot is GC-rich and repetitive, suggesting susceptibility to both replication errors and ultraviolet radiation-induced mutagenesis (Fig. 1C). The resulting truncated protein deletes part of the ATPase domain and multiple DNA-binding domains (Fig. 1D). Twenty-five of the 28 cases with the hotspot mutation were identified from other cancer types of epithelial origin, and the other three cases were melanoma (Fig. 1E).

### SRCAP-1879 truncation dominantly promotes aggressive features in a 3D cSCC model

In order to investigate the effects of these hotspot mutations, we cloned SRCAP truncated at amino acid 1879 into the pLEX-FHH backbone, which will be called SRCAP-1879. This truncation removes 42% of the total protein sequence, including the AT-hook domains and half of the bipartite ATPase catalytic domain. We expressed SRCAP-1879 on a wild-type background to mimic a somatically acquired heterozygous mutation in primary human keratinocytes. (Supplemental Fig. 1A).

**Fig. 2:**
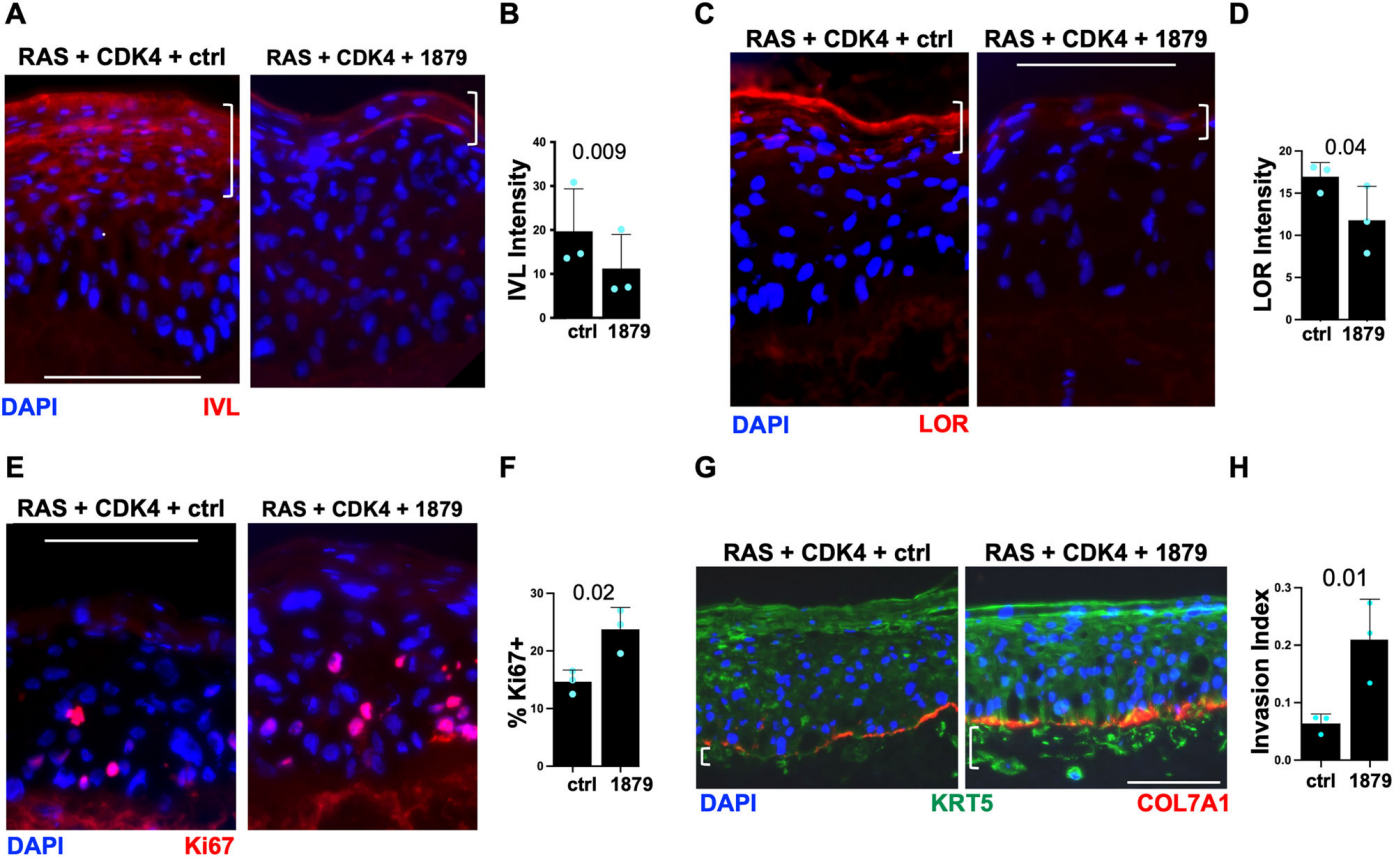
SRCAP-1879 truncation promotes aggressive features in a 3D cSCC model. Representative images and quantification of **A**, **B** involucrin, **C**, **D** loricrin, and **E**, **F** Ki67. **G** Representative images of invasion defined by KRT5 beneath the basement membrane, shown with COL7A1. **H** Quantification of invasion. White scalebars indicate 125 μM. White brackets indicate areas stained by differentiation markers or depth of invasion by KRT5 staining. All graphs display the mean and standard deviation of three biological replicates. *p*-Values calculated from *t*-tests.

To examine the role of SRCAP-1879 in cSCC, we utilized a genetically defined cSCC model driven by HRAS and CDK4 [23]. We introduced HRAS, CDK4, and either the empty vector control or SRCAP-1879 to primary human keratinocytes and then regenerated three-dimensional skin tissue. In tumors with SRCAP-1879, the differentiation markers involucrin and loricrin were significantly decreased compared to control tumors (Fig. 2A, D). Additionally, the proliferation marker Ki67 was significantly increased in the SRCAP-1879 cSCC tissue (Fig. 2E, F). Consistent with previous reports, HRAS and CDK are sufficient to drive invasion of epidermal keratinocytes into the dermis [23]. The addition of SRCAP-1879 significantly increased invasion into the dermis (Fig. 2G, H). In summary, the SRCAP mutation decreased differentiation, promoted proliferation, and increased invasion, which are usually hallmarks of more aggressive tumors.

We also examined how the SRCAP-1879 truncation affected epidermal homeostasis in tissue. The proliferation marker Ki67 was increased in the tissue with SRCAP-1879 expression relative to control (Supplemental Fig. 1B, C). The differentiation marker loricrin was increased in the SRCAP-1879 tissue (Supplemental Fig. 1D, E), and Nile red (a lipophilic dye targeting the barrier layer) was unchanged (Supplemental Fig. 1F, G). These results are consistent with prior observations that large clonal expansions of keratinocytes carrying known driver mutations frequently occur without noticeably disturbing skin morphology [8–10].

### SRCAP-1879 truncating mutation does not affect H2A.Z chromatin occupancy

**Fig. 3:**
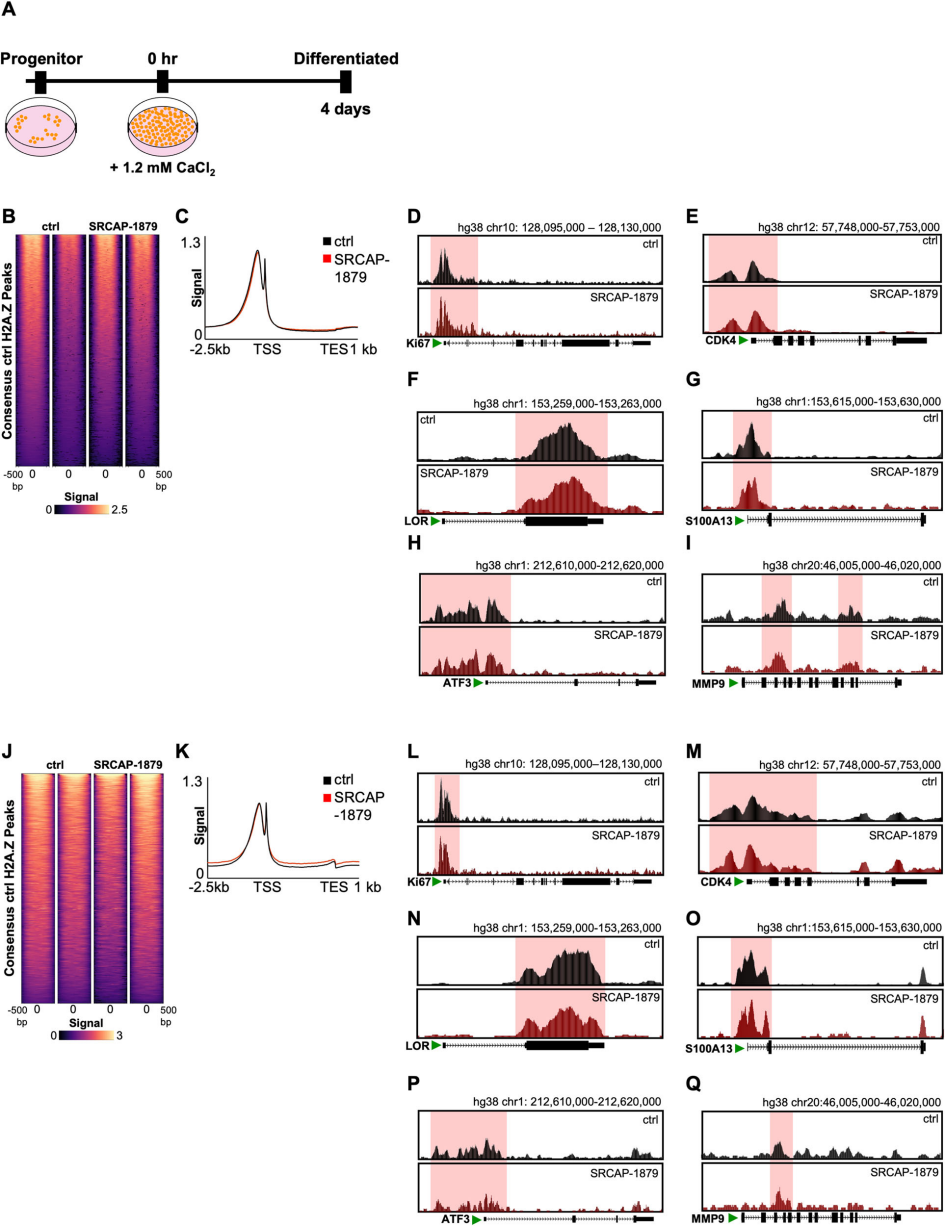
SRCAP-1879 truncation does not affect H2A.Z chromatin occupancy. **A** Diagram of keratinocyte culture for the progenitor and differentiated states. **B** Heatmap of H2A.Z chromatin occupancy in control and SRCAP-1879 in the progenitor state. **C** Average profile plot of H2A.Z occupancy at transcription start sites. **D**–**I** Representative browser tracks of H2A.Z. **J** Heatmap of H2A.Z chromatin occupancy in control and SRCAP-1879 in the differentiated state. **K** Average profile plot of H2A.Z occupancy at transcription start sites. **L**–**Q** Representative browser tracks.

SRCAP is a chromatin remodeler that deposits the histone H2A variant, H2A.Z, to modulate the chromatin state and gene expression. We investigated whether this mutation may mediate its effects by disturbing H2A.Z occupancy. Both the epidermal progenitor and differentiated states can be achieved in primary keratinocyte cultures (Fig. 3A). In the progenitor state, SRCAP-1879 expression induces no differential H2A.Z binding by ChIP-seq (Fig. 3B). We confirmed no differential H2A.Z binding using DiffBind, even with a permissive fold-change cut-off of ±1.5 and *p* < 0.05. To illustrate, we show an average profile plot of H2A.Z binding at the promoter and representative browser tracks (Fig. 3C–I). Genes of interest include proliferation markers (Ki67, CDK4), differentiation markers (LOR, S100A13, ATF3) and MMP9. We subsequently performed H2A.Z ChIP-seq in the differentiated state, and again observed no significant changes in H2A.Z binding compared to the control (Fig. 3J, K). This lack of change is further shown with representative browser tracks in (Fig. 3L–Q). We conclude that the SRCAP-1879 truncation is not sufficient to alter H2A.Z’s chromatin occupancy in the presence of wild-type SRCAP.

### SRCAP-1879 truncating mutation disrupts epidermal gene expression

**Fig. 4:**
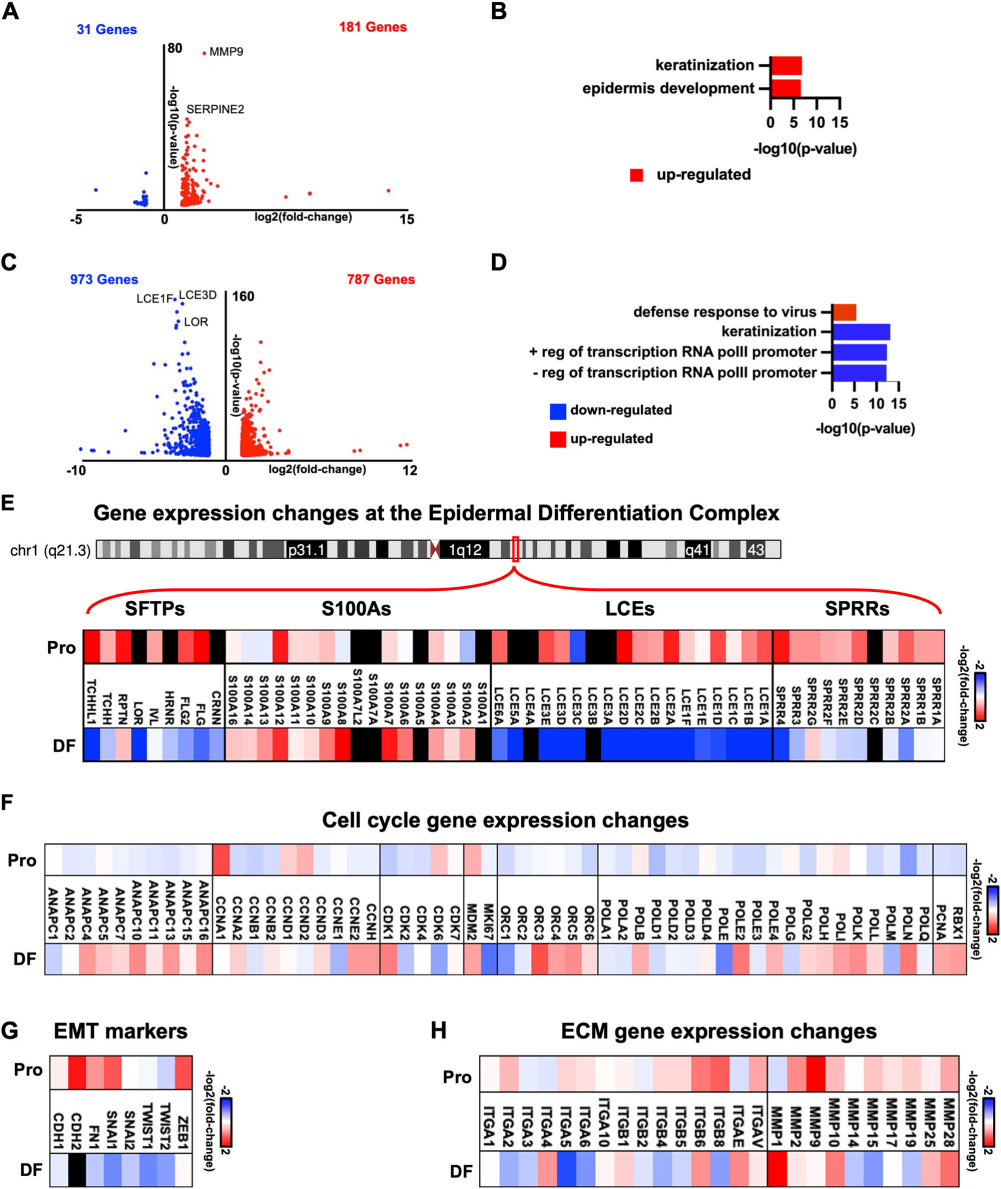
SRCAP-1879 truncation promotes gene expression changes driving cancer. **A** Volcano plot of differentially expressed genes and **B** associated GO terms in the mutant progenitor state. **C** Volcano plot of differentially expressed genes and **D** associated GO terms in the mutant progenitor state. **E** Diagram of the Epidermal Differentiation Complex and heatmap of gene expression changes at the Epidermal Differentiation Complex. **F** Heatmap of gene expression changes of proliferation genes. **G** Heatmap of gene expression changes of EMT markers. **H** Heatmap of gene expression changes affecting integrins and MMPs. All heatmaps show the log2 fold change UD 1879 relative to UD control on top and the log2 fold change DF 1879 relative to DF control on bottom.

Although SRCAP-1879 did not alter H2A.Z chromatin occupancy, we still tested for gene expression changes. First, we performed RNA-sequencing in progenitor-state keratinocytes expressing SRCAP-1879. In this cell state, SRCAP-1879 expression dysregulated only 212 genes (log2fold-change ± 1, *p* < 0.05) (Fig. 4A, Supplemental Table 1). There were too few down-regulated genes for gene ontology (GO) term analysis, but the up-regulated genes enriched GO terms such as “keratinization” and “epidermal development” (Fig. 4B), indicating that the SRCAP-1879 truncation was sufficient to induce premature expression of differentiation genes in this cell state. In addition, the matrix metallopeptidase MMP9 was among the top induced genes.

We subsequently performed RNA-sequencing in the differentiated condition (induced by 1.2 mM of CaCl2 and confluency for four days) and identified 1760 significantly changed genes (log2fold-change ± 1, *p* < 0.05) (Fig. 4C, Supplemental Table 2). Upregulated genes produced few significant GO terms, but “defense response to virus” may indicate induction of an inflammatory state. Downregulated genes were enriched for “keratinization”. The positive and negative regulation of transcription GO terms encompasses a number of transcription factors (Fig. 4D).

Both the progenitor and differentiated cells revealed abnormal expression of differentiation genes. Therefore, we examined gene expression from the epidermal differentiation complex (EDC) [24, 25], a two-megabase region of chromosome one, which contains four gene families crucial for epidermal barrier function. Three of the gene families, SFTP, LCE, and SPRR, are upregulated in the progenitor state by SRCAP-1879 compared to control (Fig. 4E). Notably, the SFTP and LCE family genes are downregulated in the differentiated state with SRCAP-1879. Cell cycle genes are generally not affected in the progenitor state, while many are upregulated in the differentiated state (Fig. 4F). This indicates that both the progenitor and differentiated keratinocytes are experiencing gene expression dysregulation with the presence of SRCAP-1879. Further, progenitors with SRCAP-1879 show an upregulation of epithelial to mesenchymal transition (EMT) markers and upregulation of motility genes (integrins and MMPs) (Fig. 4G, H). In summary, the SRCAP-1879 truncation induces transcriptional changes in multiple cellular processes associated with cancer (proliferation, differentiation, motility, and EMT) in different cell states.

To examine why the SRCAP-1879 truncation affected the differentiated state more than the progenitor state, we probed SRCAP-1879’s subcellular localization. Using an HA antibody, we stained progenitor and differentiated keratinocytes expressing SRCAP-1879 and examined its relative nuclear and cytoplasmic localization. In the differentiated state, SRCAP-1879 is enriched in the nucleus to a greater extent than in the progenitor state (Supplemental Fig. 2).

### SRCAP-1879 truncating mutation promotes migration

**Fig. 5:**
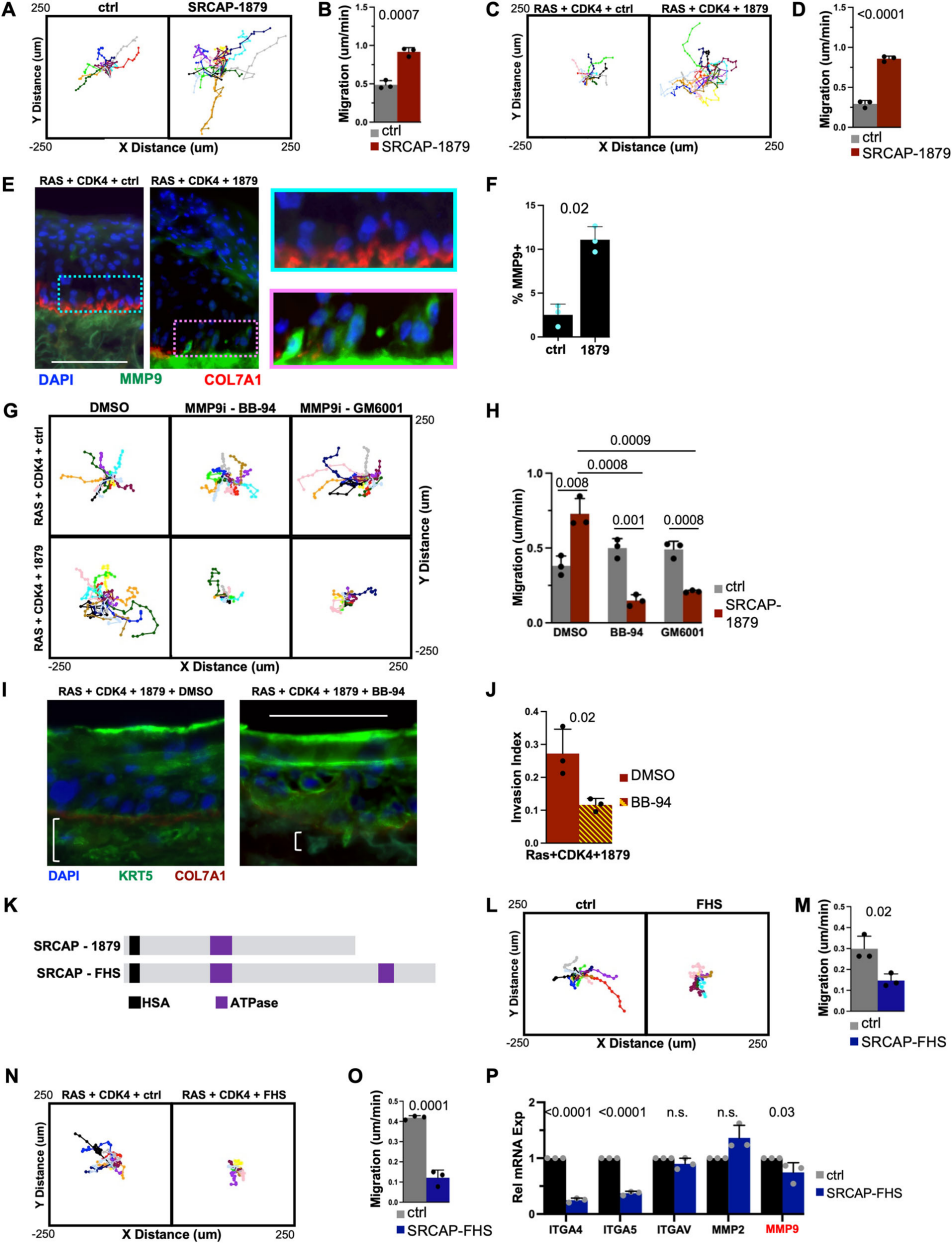
SRCAP-1879 truncation promotes migration. Representative migration tracks and quantification of in vitro cellular migration **A**, **B** in primary and **C**, **D** RAS-CDK4 expressing keratinocytes, comparing control and SRCAP-1879 mutation. **E**, **F** Representative images and quantification of MMP9. The white scale bar indicates 125 μm. Blue and pink boxes highlight basal cells, which are magnified to the right. **G**, **H** Representative migration tracks and quantification of migration in RAS-CDK4 expressing keratinocytes with MMP inhibition. **I**, **J** Representative images and quantification of invasion with MMP inhibition, in Ras-CDK4 epidermal tissue model with or without co-expression of SRCAP-1879. **K** Diagram comparing SRCAP-1879 and SRCAP-FHS proteins. Representative migration tracks and quantification of in vitro cellular migration **L**, **M** in primary and **N**, **O** in RAS-CDK4 expressing keratinocytes, comparing control and SRCAP-FHS mutation. **P** rt-qPCR of integrins and MMPs comparing control and SRCAP-FHS in primary keratinocytes. All migration tracks show representative tracks of 15 cells. All bar graphs display the mean and standard deviation of three biological replicates. *p*-Values calculated from t-tests.

Because invasion and metastasis are the most dangerous aspects of cSCC, we further probed the increased invasion induced by the SRCAP-1879 truncation. SRCAP-1879 in primary human keratinocytes increased migration relative to control (Fig. 5A, B). Because progenitor keratinocytes reside on a collagen-rich basement membrane [26], we coated plates in collagen, but the presence of collagen did not alter migration dynamics (Supplemental Fig. 3A, B). We next examined migration in the RAS-CDK4 keratinocytes with SRCAP-1879, and saw a similar increase in migration, which was also not affected by collagen-coating (Fig. 5C, D, Supplemental Fig. 3C, D). To determine if SRCAP-1879 affects cell migration in other cancer types, we performed the migration assay using A-253 (salivary gland carcinoma), CAL 27 (tongue SCC), and HCT 116 (colorectal carcinoma) cells, and in every case, SRCAP-1879 significantly increased migration (Supplementary Fig. 3E–J).

Our RNA-sequencing data indicated that SRCAP-1879 truncation strongly increases the expression of MMP9. Therefore, we stained the RAS-CDK4 cSCC tissues for MMP9 and observed a significant increase in MMP9+ cells near the basement membrane (Fig. 5E, F). Therefore, we tested two MMP inhibitors in RAS-CDK4 expressing keratinocytes. As before, SRCAP-1879 increases invasion compared to the control in the DMSO control. The MMP inhibitors did not significantly alter the migration of the control, but did significantly decrease the migration of the keratinocytes expressing SRCAP-1879 (Fig. 5G, H). Consistently, in epidermal tissue with RAS-CDK4 as well as SRCAP-1879 expression, MMP inhibition significantly decreased dermal invasion (Fig. 5I, J), suggesting the MMP9 activation by SRCAP-1879 is a critical step in promoting cSCC invasion.

Finally, we wanted to compare the SRCAP-1879 cancer hotspot mutation to a known pathogenic SRCAP mutation. Therefore, we cloned the most common floating harbor syndrome (FHS) mutation (SRCAP-FHS), which truncates the AT-hooks (Fig. 5K) and performed the migration assay. In contrast to SRCAP-1879, SRCAP-FHS decreased migration in both primary and RAS-CDK4 expressing keratinocytes (Fig. 5L–O), which is consistent with a previous report [13]. Although integrins and MMPs were upregulated by SRCAP-1879, these genes were either unchanged or downregulated by SRCAP-FHS (Fig. 5P). SRCAP-FHS also upregulated differentiation genes in progenitors consistent with SRCAP-1879, and downregulated cell cycle genes in progenitors (Supplemental Fig. 4).

## Discussion

In this study, we first noted that SRCAP is frequently mutated in keratinocyte carcinomas. Using pan-cancer data, we identified the mutational hotspot in SRCAP at amino acid 1879. This SRCAP-1879 truncation impaired epidermal differentiation, increased proliferation, and promoted invasion in an HRAS-CDK4-driven cSCC model. We also observed upregulation of integrins and MMPs, which are associated with cancer invasion [27, 28].

The cancer hotspot mutation altered gene expression without changing H2A.Z chromatin occupancy in the presence of wild-type SRCAP. A previous report showed that H2A.Z chromatin occupancy is disturbed in Floating Harbor Syndrome [13]. However, the canonical exon 33/34 FHS truncations do not affect the ATPase domain like the SRCAP-1879 truncation. Further, only minor skin symptoms have been reported in FHS, and increased malignancy has not been reported [18, 29]. While we targeted a mutational hotspot, truncating and missense mutations occur across the full length of SRCAP, and those mutations may induce additional phenotypes.

Since the SRCAP-1879 truncation does not alter H2A.Z occupancy, it suggests that the pro-cancer effects of the cancer hotspot mutation may be H2A.Z independent. SRCAP is the scaffold of a multi-protein chromatin remodeling complex that participates in critical processes including DNA repair [30, 31], and mitosis [32]. Further, SRCAP has been shown to interact with lineage-specific and general transcription factors, including p63, PCID2, and the REST complex [11, 15, 33]. The truncated protein could sequester necessary proteins away from the wild-type SRCAP complex, lose its ability to interact with appropriate protein partners, or inappropriately interact with proteins. In addition, the cytoplasmic localization of SRCAP-1879, especially in the progenitor-state keratinocytes, suggests that this truncation may also dysregulate cytoplasmic proteins beyond the well-recognized nuclear processes. Further investigation is needed to define a precise molecular mechanism behind the promotion of aggressive cSCC features that we observed.

This SRCAP-1879 hotspot truncation has previously been noted in a colon cancer DNA-sequencing study [34], although the functional significance of this mutation remained unclear. Our characterization of this hotspot mutation, leveraging primary human keratinocytes and the Ras-CDK4 cSCC model, demonstrates that the SRCAP-1879 truncation is sufficient to dysregulate gene expression in a cell-state-dependent manner and promote cancer invasion with the induction of MMP9. Further supporting a broader role of SRCAP-1879 in cancer (especially in carcinoma) progression, we found that the expression of SRCAP-1879 was sufficient to promote cell motility in several cancer cell lines, including the HCT116 colorectal carcinoma cell line, the A-253 salivary gland carcinoma cell line, and the CAL 27 tongue squamous cell carcinoma cell line. While the functional characterizations for SRCAP-1879’s roles in cancer progression in this study focus on cancer invasion, our RNA-seq data demonstrate that SRCAP-1879 influences additional processes such as differentiation, proliferation and extracellular matrix, suggesting that SRCAP-1879 can promote cancer progression through dysregulating other processes in addition to invasion. Furthermore, we found that SRCAP-1879’s downstream genes and subcellular localization exhibit cell-state specificity. For example, MMP9 is highly induced in keratinocyte culture in the progenitor state, but not in the differentiation state. SRCAP-1879’s subcellular localization also significantly increases in the nuclear compartment in the differentiation state as compared to the progenitor state. Future work determining how SRCAP-1879’s subcellular localization is regulated, and how cytoplasmic and nuclear fractions of SRCAP-1879 influence gene expression, can inform therapeutic design targeting this hotspot truncating mutation. In addition, it will be interesting in future studies to contrast the functions and regulation between SRCAP-1879 and SRCAP-FHS, and identify other mutation sites in SRCAP with oncogenic potential.

## Materials and methods

### Keratinocyte culture

Primary human epidermal keratinocytes were isolated and pooled from a minimum of six fresh, de-identified foreskins obtained from Northwestern Skin Biology & Diseases Resource-Based Center (SBDRC). Keratinocytes were maintained in a 1:1 mixture of Keratinocyte-SFM (Life Technologies, Carlsbad, CA) and Medium 154 (Life Technologies). Keratinocytes were seeded at confluency with 1.2 mM CaCl_2_ to induce differentiation.

### Cancer cell lines

A-253 (ATCC HTB-41), CAL 27 (ATCC CRL-2095), and HCT 116 (ATCC CCL-247) were purchased from ATCC. A-253 and CAL 27 were cultured in DMEM (Gibco, Waltham, MA), and HCT 116 was cultured in McCoy’s 5A media (Gibco). All were supplemented with 10% fetal bovine serum (HyClone, Logan, UT).

### Retro/lentiviral production

Phoenix and HEK293T cells were cultured in DMEM (Gibco) supplemented with 10% fetal bovine serum (HyClone, Logan, UT). Transfection was performed with Lipofectamine 3000 Transfection Reagent (Invitrogen, Carlsbad, CA). Virus was collected 48- and 72-h post-transfection and cleared of cellular debris by centrifugation.

### Transduction

Keratinocytes or cancer cell lines were centrifuged with virus and polybrene (20 μg/mL, Sigma Aldrich, Burlington, MA) for one hour at 32 °C at 1250 RPM. If a selection marker was present, puromycin (2 μg/mL, ThermoFisher, Waltham, MA) selection was initiated 24 h post-infection for 24 h.

### Plasmid construction

Gene expression constructs were generated with In-fusion HD Cloning Kit (Takara, San Jose, CA) and expressed from the pLEX-FHH-puro backbone (Addgene 120569, Watertown, MA) [35]. The empty vector was used as the control in this study. Cloning primers were generated using the In-Fusion Primer Design Tool (Takara). Cancer mutation forward primer CCACCATCATGGATCCATGCAGAGCAGCCCCTCC and reverse primer TAGACTCGAGCGGCCGCCTAGGTGGGGGGGGGCTG. Floating Harbor Syndrome forward primer CCACCATCATGGATCCATGCAGAGCAGCCCCTCC and reverse primer TAGACTCGAGCGGCCGCTCAAGGCCTAGGTGCTGG. Plasmid construction of ER-HRas^v12^ and CDK4 overexpression was described in [23].

### Western Blot

Keratinocytes were collected in urea lysis buffer, which contains: 8 M urea, 10% BME, 65 mM CHAPS, 40 mM Tris, and 2.5 mM EDTA. 30 μg of protein was separated by SDS-PAGE and transferred to a nitrocellulose membrane. Membranes were blocked with Intercept Protein Free Blocking Buffer (Li-COR, Lincoln, NE). Primary antibodies (HA-Tag Cell Signaling #3724 and Lamin A/C Santa Cruz sc-376248) were incubated overnight at 4˚C. Secondary antibodies (IRDye 680LT goat anti-mouse or IRDye 800CW goat anti-rabbit LI-COR) were diluted 1:20,000 and incubated for up to two hours at room temperature. Blots were scanned on a Li-COR Odyssey (Li-COR). A representative of three biological replicates is shown.

### Migration assay

Keratinocytes or cell lines were seeded onto 12-well plates. For collagen coating, collagen from calf skin (Sigma Aldrich C8919-20ML) was added at a concentration of 0.1 mg/mL overnight at 37 °C. The excess was removed, and plates were allowed to dry before seeding. HRAS-CDK4 cells were induced with 100 nM tamoxifen for 48 h prior to imaging. Matrix metalloprotease inhibitors BB-94 and GM6001 were added for 24 h prior to imaging at a concentration of 50 μM. Images were acquired on an Evos FL Auto 2 utilizing the on-stage live incubator (Thermo Fisher Scientific, Waltham, MA. Images were taken every 15 min for 4 h. Migration was quantified using ImageJ’s Manual Tracking plug-in for 15 cells per condition. Graphs show the mean of three biological replicates with statistics derived from a student’s *t*-test, where p-value < 0.05 was considered significant.

### Organotypic culture

De-vitalized split-thickness human skin dermis was obtained from The New York Firefighters Skin Bank. The use of human dermis from de-identified donors has been approved by Northwestern’s IRB. Dermis was suspended on a physical support, and the bottom was coated with Matrigel (Corning, 354234, Corning, NY) on the bottom. In total, 750,000 keratinocytes were seeded per dermis with FAD media for 24 h then switched to a 50% FAD and 50% E media mixture for the remainder of culture time. Cultures were maintained at a liquid/air interface for 6 days. For the cSCC model, keratinocytes were allowed to stratify for 4 days before H-Ras expression was induced with 100 nM tamoxifen and cultures were grown for 6 more days, for a total duration of 10-day regeneration. For matrix metalloprotease inhibition, 50 μM BB-94 was added after stratification through day 10. Tissue was embedded in OCT. Tissue was fixed with either 10% formalin for ten minutes or a 1:1 mixture of cold acetone and methanol for six minutes. Tissue was blocked in 5% goat serum and 0.5% Triton X-100. The tissue was incubated overnight at 4 °C with the primary antibody. The secondary antibody was incubated for two hours at room temperature. The tissue was incubated with Hoechst for five minutes. Nile Red (Sigma Aldrich) was applied to the tissue for ten minutes. Images were acquired on an Evos FL Auto 2 (Invitrogen) and quantified using ImageJ.

Antibodies used were involucrin (Proteintech 28462-1-AP, 1:500, Rosemont, IL), loricrin (BioLegend 905104, 1:500, San Diego, CA), MMP9 (Cell Signaling 13667, 1:500, Danvers, MA), Ki67 (Cell Signaling 9129, 1:500), KRT5 (Biolegend 905501, 1:500), and COL7A1 (Santa Cruz sc-33710, 1:200, Dallas, TX). Secondary antibodies were Alexa Fluor 488 goat anti-rabbit IgG (Life Technologies A11304, 1:500) and Alexa Fluor 555 goat anti-mouse IgG (Life Technologies A21422, 1:500).

For quantification, the mean intensity of the Nile red or differentiation markers, the antibody-positive area was quantified for five non-overlapping fields per biological replicate. Ki67-positive nuclei or MMP9-positive cells were manually counted in five fields per biological replicate. The invasion index was calculated by dividing the area of invasion by the total dermis area. Invasion was defined as the KRT5+ region in the dermal compartment, defined by the COL7A1 boundary. Data are displayed as mean ± standard deviation of three biological replicates. p-values were calculated by Student’s *t*-test.

### rt-qPCR

RNA was extracted with the Quick-RNA Miniprep Kit (Zymo Research, Irvine, CA). SuperScript VILO cDNA Synthesis Kit (Thermo Fisher) was used to make cDNA. qRT-PCR was performed with PowerUp SYBR Green Master Mix (Thermo Fisher) with a QuantStudio 3 Real-Time PCR System (Thermo Fisher). Primer sequences are listed in Supplemental Table 3. Ct values were normalized to 18S. Data are displayed as mean ± standard deviation of three biological replicates. *p*-values were calculated by Student’s *t*-test.

### RNA sequencing

RNA was extracted with the Quick-RNA MiniPrep kit, including the DNase I treatment step (Zymo Research). Libraries were prepared using the NEBNext Poly(A) mRNA Magnetic Isolation Module (New England Biolabs, Ipswich, MA) and the NEBNext Ultra II Directional RNA Library Prep Kit for Illumina (New England BioLabs). Northwestern University’s NUSeq core facility generated single-end 50 base-pair reads on an Illumina HiSeq 4000.

For data processing, we utilized Nextflow [36]. Read quality control was performed with FastQC. Adaptors were removed with Trimgalore, and reads were aligned to hg38 by STAR [37]. Gene expression was quantified using Salmon [38]. In R, DESeq2 [39] was used to calculate differential expression. Genes were considered differentially expressed if log_2_(fold change) ± 1 and *p* value < 0.05. Gene ontology using lists of differentially expressed genes analysis was performed with DAVID, considered significant if FDR < 0.05 [40].

### ChIP-sequencing

Our ChIP-seq protocol has been described in detail previously [41]. Briefly, keratinocytes were fixed using 1% formaldehyde. Fixation was quenched with 0.125 M glycine. Nuclei were extracted and lysed. Lysate was sonicated with a Bioruptor Pico sonicator (Diagenode, Denville, NJ) and then incubated overnight with H2A.Z antibody (Active Motif 39113, Carlsbad, CA) and Protein G Dynabeads (Thermo Fisher). The beads were washed before DNA was eluted at 67 °C overnight. The eluate was incubated with RNase A at 37 °C for 1 h, then with proteinase K at 55 °C for 1 h. DNA was purified using the ChIP DNA Clean and Concentrator Kit (Zymo Research). DNA concentrations were measured with the Qubit dsDNA HS assay (Thermo Fisher). Libraries were prepared with NEBNext Ultra II DNA Library Prep Kit for Illumina (New England BioLabs). The Northwestern University NUSeq Core Facility sequenced libraries as single-end 50 base pair reads on an Illumina HiSeq 4000.

Analysis utilized Nextflow, FastQC, and Trimgalore as described above. Reads were aligned to hg38 with BWA-MEM [42] under the default settings. MACS2 [43] was used to call narrow peaks, *p* < 0.001. DiffBind [44] was used to calculate differential binding, which was considered significant if log_2_(fold change) was ±1 and *p* value < 0.05. Bedtools and DeepTools [45] were utilized for data visualization.

### Publicly available data

All data regarding frequency and location of genetic alterations in cancer were retrieved from cBioPortal.org [22].

## Supplementary information

supplemental figures

Data Set 1

Data Set 2

Data Set 3

## Data Availability

The ChIP-sequencing and RNA-sequencing data generated in this study are available at GSE292167 and GSE292168.

## References

[1] Siegel RL, Miller KD, Wagle NS, Jemal A. Cancer statistics, 2023. CA Cancer J Clin. 2023;73:17–48. 36633525 10.3322/caac.21763

[2] Muzic JG, Schmitt AR, Wright AC, Alniemi DT, Zubair AS, Olazagasti Lourido JM, et al. Incidence and trends of basal cell carcinoma and cutaneous squamous cell carcinoma: a population-based study in Olmsted County, Minnesota, 2000 to 2010. Mayo Clin Proc. 2017;92:890–8. 28522111 10.1016/j.mayocp.2017.02.015 PMC5535132

[3] Mansouri B, Housewright CD. The treatment of actinic keratoses-the rule rather than the exception. JAMA Dermatol. 2017;153:1200. 28975200 10.1001/jamadermatol.2017.3395

[4] Fuchs E. Scratching the surface of skin development. Nature. 2007;445:834–42. 17314969 10.1038/nature05659 PMC2405926

[5] Chalmers ZR, Connelly CF, Fabrizio D, Gay L, Ali SM, Ennis R, et al. Analysis of 100,000 human cancer genomes reveals the landscape of tumor mutational burden. Genome Med. 2017;9:34. 28420421 10.1186/s13073-017-0424-2 PMC5395719

[6] South AP, Purdie KJ, Watt SA, Haldenby S, den Breems N, Dimon M, et al. NOTCH1 mutations occur early during cutaneous squamous cell carcinogenesis. J Invest Dermatol. 2014;134:2630–8. 24662767 10.1038/jid.2014.154 PMC4753672

[7] Bonilla X, Parmentier L, King B, Bezrukov F, Kaya G, Zoete V, et al. Genomic analysis identifies new drivers and progression pathways in skin basal cell carcinoma. Nat Genet. 2016;48:398–406. 26950094 10.1038/ng.3525

[8] Wang Y, Masaki T, Khan SG, Tamura D, Kuschal C, Rogers M, et al. Four-dimensional, dynamic mosaicism is a hallmark of normal human skin that permits mapping of the organization and patterning of human epidermis during terminal differentiation. PLoS ONE. 2018;13:e0198011. 29897937 10.1371/journal.pone.0198011 PMC5999106

[9] Yizhak K, Aguet F, Kim J, Hess JM, Kubler K, Grimsby J. RNA sequence analysis reveals macroscopic somatic clonal expansion across normal tissues. Science. 2019;380:eabq4567 10.1126/science.aaw0726 PMC7350423 31171663

[10] Martincorena I, Roshan A, Gerstung M, Ellis P, Van Loo P, McLaren S, et al. Tumor evolution. High burden and pervasive positive selection of somatic mutations in normal human skin. Science. 2015;348:880–6. 25999502 10.1126/science.aaa6806 PMC4471149

[11] Ye B, Yang L, Qian G, Liu B, Zhu X, Zhu P, et al. The chromatin remodeler SRCAP promotes self-renewal of intestinal stem cells. EMBO J. 2020;39:e103786. 32449550 10.15252/embj.2019103786 PMC7327502

[12] Faast R, Thonglairoam V, Schulz TC, Beall J, Wells JR, Taylor H, et al. Histone variant H2A.Z is required for early mammalian development. Curr Biol. 2001;11:1183–7. 11516949 10.1016/s0960-9822(01)00329-3

[13] Greenberg RS, Long HK, Swigut T, Wysocka J. Single amino acid change underlies distinct roles of H2A.Z subtypes in human syndrome. Cell. 2019;178:1421–36 e24. 31491386 10.1016/j.cell.2019.08.002 PMC7103420

[14] Creyghton MP, Markoulaki S, Levine SS, Hanna J, Lodato MA, Sha K, et al. H2AZ is enriched at polycomb complex target genes in ES cells and is necessary for lineage commitment. Cell. 2008;135:649–61. 18992931 10.1016/j.cell.2008.09.056 PMC2853257

[15] Ye B, Liu B, Yang L, Huang G, Hao L, Xia P, et al. Suppression of SRCAP chromatin remodelling complex and restriction of lymphoid lineage commitment by Pcid2. Nat Commun. 2017;8:1518. 29138493 10.1038/s41467-017-01788-7 PMC5686073

[16] Zhao B, Chen Y, Jiang N, Yang L, Sun S, Zhang Y, et al. Znhit1 controls intestinal stem cell maintenance by regulating H2A.Z incorporation. Nat Commun. 2019;10:1071. 30842416 10.1038/s41467-019-09060-w PMC6403214

[17] Xu Y, Ayrapetov MK, Xu C, Gursoy-Yuzugullu O, Hu Y, Price BD. Histone H2A.Z controls a critical chromatin remodeling step required for DNA double-strand break repair. Mol Cell. 2012;48:723–33. 23122415 10.1016/j.molcel.2012.09.026 PMC3525728

[18] Nikkel SM, Dauber A, de Munnik S, Connolly M, Hood RL, Caluseriu O, et al. The phenotype of Floating-Harbor syndrome: clinical characterization of 52 individuals with mutations in exon 34 of SRCAP. Orphanet J Rare Dis. 2013;8:63. 23621943 10.1186/1750-1172-8-63 PMC3659005

[19] Zhang H, Li S, Zhou R, Dong T, Zhang X, Yu M, et al. SRCAP complex promotes lung cancer progression by reprograming the oncogenic transcription of Hippo-YAP/TAZ signaling pathway. Cancer Lett. 2024;585:216667. 38280479 10.1016/j.canlet.2024.216667

[20] Chen CW, Zhang L, Dutta R, Niroula A, Miller PG, Gibson CJ, et al. SRCAP mutations drive clonal hematopoiesis through epigenetic and DNA repair dysregulation. Cell Stem Cell. 2024;31:275–7. 38306995 10.1016/j.stem.2024.01.001 PMC10981498

[21] Valimaki N, Jokinen V, Cajuso T, Kuisma H, Taira A, Dagnaud O, et al. Inherited mutations affecting the SRCAP complex are central in moderate-penetrance predisposition to uterine leiomyomas. Am J Hum Genet. 2023;110:460–74. 36773604 10.1016/j.ajhg.2023.01.009 PMC10027472

[22] Cerami E, Gao J, Dogrusoz U, Gross BE, Sumer SO, Aksoy BA, et al. The cBio cancer genomics portal: an open platform for exploring multidimensional cancer genomics data. Cancer Discov. 2012;2:401–4. 22588877 10.1158/2159-8290.CD-12-0095 PMC3956037

[23] Lazarov M, Kubo Y, Cai T, Dajee M, Tarutani M, Lin Q, et al. CDK4 coexpression with Ras generates malignant human epidermal tumorigenesis. Nat Med. 2002;8:1105–14. 12357246 10.1038/nm779

[24] Oh IY, de Guzman Strong C. The molecular revolution in cutaneous biology: EDC and locus control. J Invest Dermatol. 2017;137:e101–e4. 28411839 10.1016/j.jid.2016.03.046 PMC6400479

[25] Kypriotou M, Huber M, Hohl D. The human epidermal differentiation complex: cornified envelope precursors, S100 proteins and the ‘fused genes’ family. Exp Dermatol. 2012;21:643–9. 22507538 10.1111/j.1600-0625.2012.01472.x

[26] Rousselle P, Laigle C, Rousselet G. The basement membrane in epidermal polarity, stemness, and regeneration. Am J Physiol Cell Physiol. 2022;323:C1807–C22. 36374168 10.1152/ajpcell.00069.2022

[27] Baster Z, Russell L, Rajfur Z. A review of talin- and integrin-dependent molecular mechanisms in cancer invasion and metastasis. Int J Mol Sci. 2025;26:1798 40076426 10.3390/ijms26051798 PMC11899650

[28] Rashid ZA, Bardaweel SK. Novel matrix metalloproteinase-9 (MMP-9) inhibitors in cancer treatment. Int J Mol Sci. 2023;24:12133 37569509 10.3390/ijms241512133 PMC10418771

[29] Ercoskun P, Yuce-Kahraman C. Novel findings in floating-harbor syndrome and a mini-review of the literature. Mol Syndromol. 2021;12:52–6. 33776628 10.1159/000512050 PMC7983618

[30] Dong S, Han J, Chen H, Liu T, Huen MSY, Yang Y, et al. The human SRCAP chromatin remodeling complex promotes DNA-end resection. Curr Biol. 2014;24:2097–110. 25176633 10.1016/j.cub.2014.07.081

[31] Chen CW, Zhang L, Dutta R, Niroula A, Miller PG, Gibson CJ, et al. SRCAP mutations drive clonal hematopoiesis through epigenetic and DNA repair dysregulation. Cell Stem Cell. 2023;30:1503–19 e8. 37863054 10.1016/j.stem.2023.09.011 PMC10841682

[32] Messina G, Prozzillo Y, Monache FD, Santopietro MV, Dimitri P. Evolutionary conserved relocation of chromatin remodeling complexes to the mitotic apparatus. BMC Biol. 2022;20:172. 35922843 10.1186/s12915-022-01365-5 PMC9351137

[33] Gallant-Behm CL, Ramsey MR, Bensard CL, Nojek I, Tran J, Liu M, et al. DeltaNp63alpha represses anti-proliferative genes via H2A.Z deposition. Genes Dev. 2012;26:2325–36. 23019126 10.1101/gad.198069.112 PMC3475804

[34] Moon SW, Mo HY, Choi EJ, Yoo NJ, Lee SH. Cancer-related SRCAP and TPR mutations in colon cancers. Pathol Res Pract. 2021;217:153292. 33307343 10.1016/j.prp.2020.153292

[35] Kovalski JR, Bhaduri A, Zehnder AM, Neela PH, Che Y, Wozniak GG, et al. The functional proximal proteome of oncogenic Ras includes mTORC2. Mol Cell. 2019;73:830–44 e12. 30639242 10.1016/j.molcel.2018.12.001 PMC6386588

[36] Di Tommaso P, Chatzou M, Floden EW, Barja PP, Palumbo E, Notredame C. Nextflow enables reproducible computational workflows. Nat Biotechnol. 2017;35:316–9. 28398311 10.1038/nbt.3820

[37] Dobin A, Davis CA, Schlesinger F, Drenkow J, Zaleski C, Jha S, et al. STAR: ultrafast universal RNA-seq aligner. Bioinformatics. 2013;29:15–21. 23104886 10.1093/bioinformatics/bts635 PMC3530905

[38] Patro R, Duggal G, Love MI, Irizarry RA, Kingsford C. Salmon provides fast and bias-aware quantification of transcript expression. Nat Methods. 2017;14:417–9. 28263959 10.1038/nmeth.4197 PMC5600148

[39] Love MI, Huber W, Anders S. Moderated estimation of fold change and dispersion for RNA-seq data with DESeq2. Genome Biol. 2014;15:550. 25516281 10.1186/s13059-014-0550-8 PMC4302049

[40] Sherman BT, Hao M, Qiu J, Jiao X, Baseler MW, Lane HC, et al. DAVID: a web server for functional enrichment analysis and functional annotation of gene lists (2021 update). Nucleic Acids Res. 2022:W216-21. 10.1093/nar/gkac194 PMC9252805 35325185

[41] Droll SH, Zhang BJ, Levine MC, Xue C, Ho PJ, Bao X. CASZ1 Is Essential for skin epidermal terminal differentiation. J Invest Dermatol. 2024;144:2029–38. 38458428 10.1016/j.jid.2024.02.014 PMC11344692

[42] Li H. Aligning sequence reads, clone sequences and assembly contigs with BWA-MEM. 2013. Preprint at arXiv 10.48550/arXiv.1303.3997.

[43] Zhang Y, Liu T, Meyer CA, Eeckhoute J, Johnson DS, Bernstein BE, et al. Model-based analysis of ChIP-Seq (MACS). Genome Biol. 2008;9:R137. 18798982 10.1186/gb-2008-9-9-r137 PMC2592715

[44] Ross-Innes CS, Stark R, Teschendorff AE, Holmes KA, Ali HR, Dunning MJ, et al. Differential oestrogen receptor binding is associated with clinical outcome in breast cancer. Nature. 2012;481:389–93. 22217937 10.1038/nature10730 PMC3272464

[45] Ramirez F, Ryan DP, Gruning B, Bhardwaj V, Kilpert F, Richter AS, et al. deepTools2: a next generation web server for deep-sequencing data analysis. Nucleic Acids Res. 2016;44:W160–5. 27079975 10.1093/nar/gkw257 PMC4987876

