# Supplementary material for: A dominant SRCAP truncating mutation promotes squamous cell carcinoma progression
Source: Oncogenesis. 2025 Aug 26;14(1):33. doi: 10.1038/s41389-025-00576-z (PMC12381255; doi:10.1038/s41389-025-00576-z)
Supplement: Supplementary file 1 — supplemental figures [file 41389_2025_576_MOESM1_ESM.pdf]

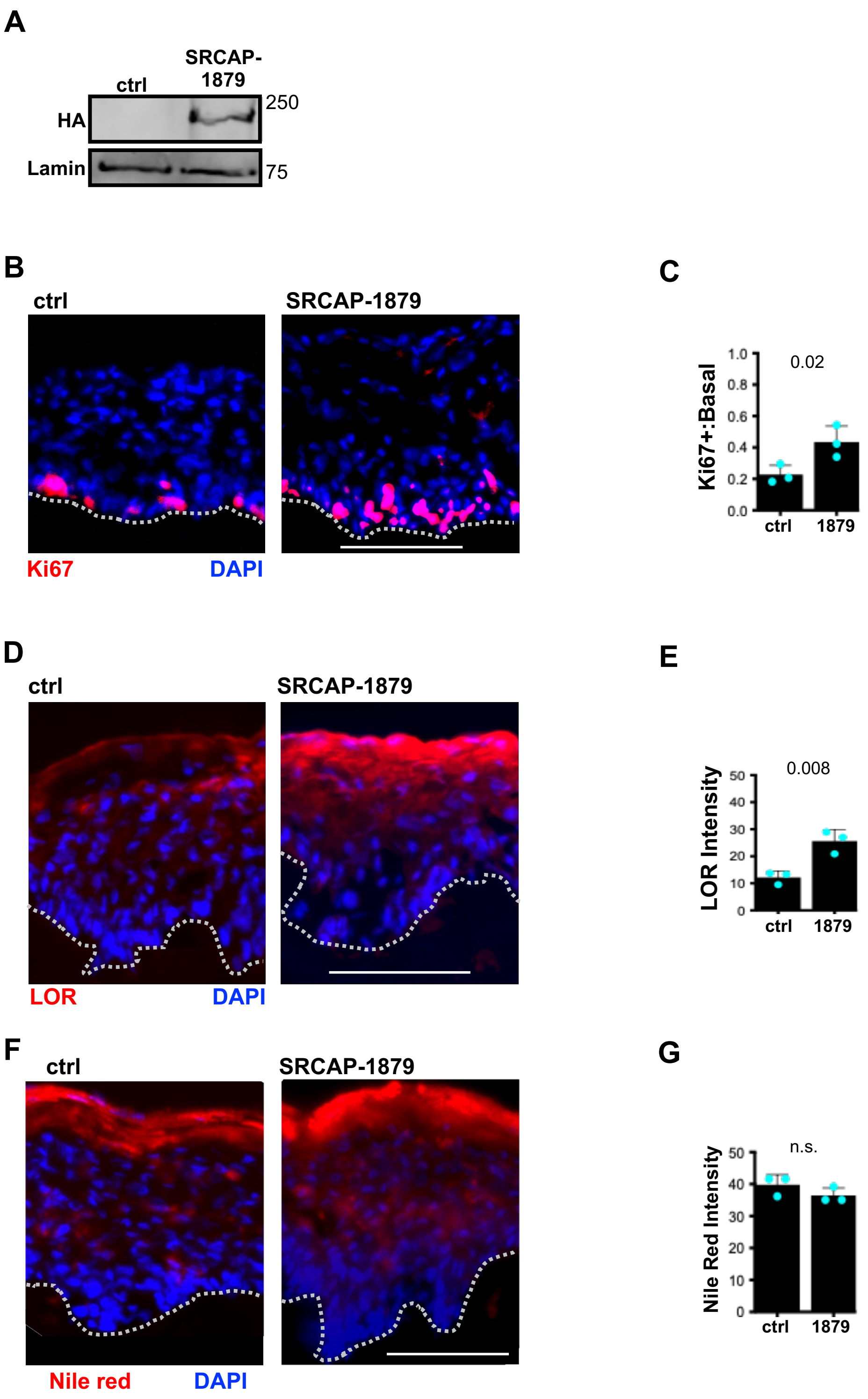

Supplemental Fig. 1: SRCAP-1879 truncation disrupts epidermal homeostasis. A Representative western blot of SRCAP-1879 expression. Representative images and quantification of **B-C** Ki67 **D-E** LOR, and **F-G** Nile red staining, in epidermal tissue regenerated using primary human keratinocytes expressing SRCAP-1879 versus control. White scalebars indicate 125 uM. All graphs display mean and standard deviation of three biological replicates. p-values calculated from t-tests.

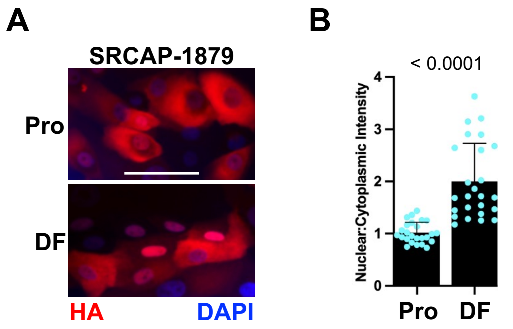

Supplemental Fig. 2: SRCAP-1879 truncation exhibits dynamic localization. **A** Representative images of SRCAP-1879 localization in primary human keratinocytes culture in progenitor state (Pro) or differentiation state (DF) detected by HA antibody, and **B** quantification of nuclear:cytoplasmic ratio of SRCAP-1879 localization in these two states.

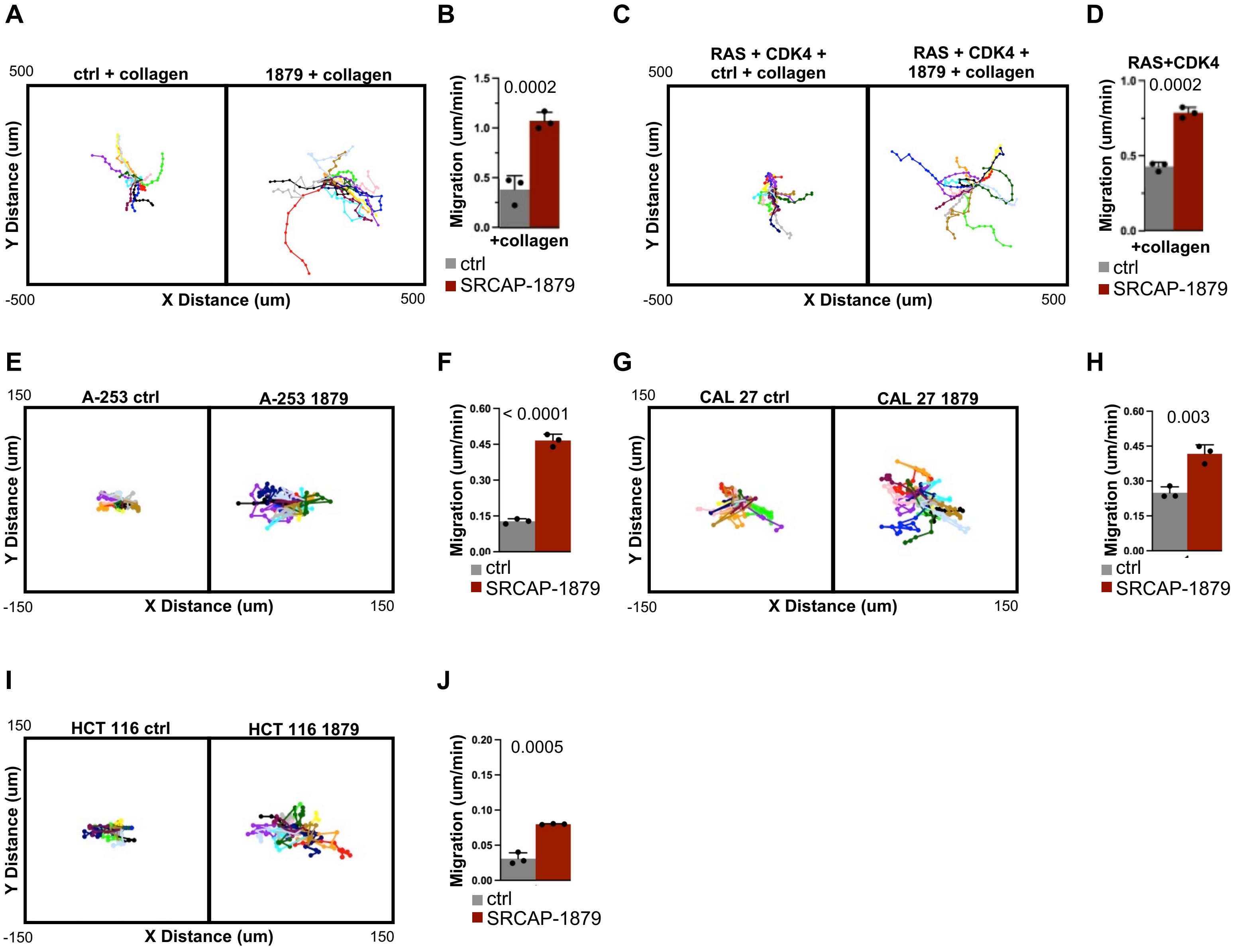

Supplemental Fig. 3: SRCAP-1879 truncation promotes cell motility. Representative migration tracks and quantification of migration on collagen coated wells for **A-B** Primary keratinocyte and **C-D** Ras-CDK4 keratinocytes. Representative migration tracks and quantification in **E-F** A-253, **G-H** CAL 27, and **I-J** HCT 116 cells.

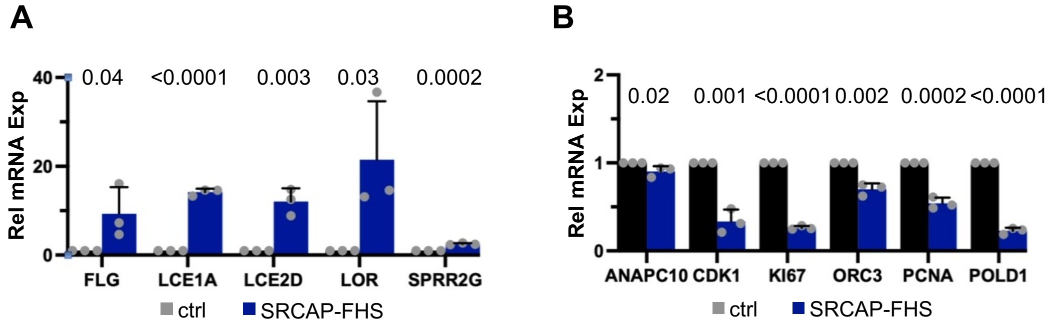

Supplemental Fig. 4: SRCAP-FHS truncation also influences keratinocyte proliferation and differentiation gene expression. rt-qPCR of representative proliferation and differentiation genes. All bar graphs show the mean ± SD of three biological replicates with p-values derived from student's t-tests.
